# Match of the Bimaxillary Basal Bone Arches and Its Variations among Individuals

**DOI:** 10.1155/2021/9625893

**Published:** 2021-11-05

**Authors:** Wenqian Chen, Hao Zeng, Luna Sun, Qiuping Xu, Zhenxue Chen, Yunhan Sun, Qi Jia, Chengyun Liu, Jing Guo

**Affiliations:** ^1^Department of Orthodontics, School and Hospital of Stomatology, Cheeloo College of Medicine, Shandong University & Shandong Key Laboratory of Oral Tissue Regeneration & Shandong Engineering Laboratory for Dental Materials and Oral Tissue Regeneration, Jinan, China; ^2^Gregory and Paula Chow Center for Economic Research, Xiamen University, Xiamen, China; ^3^School of Control Science and Engineering, Shandong University, Jinan, China; ^4^School of Stomatology, Shandong First Medical University, Tai'an, Shandong 271016, China; ^5^Savaid Stomatology School, Hangzhou Medical College, Hangzhou, China; ^6^Ningbo Stomatology Hospital, Zhejiang, China

## Abstract

**Introduction:**

This study is aimed at illustrating the bimaxillary basal bone contours, to clarify the match of the basal bone arches of the upper and lower, especially the posterior segments, including the second molar and retromolar region.

**Methods:**

Based on 100 cone-beam computed tomography (CBCT) images (50 males and 50 females), we obtained 100 pairs of basal bone arches, which were the horizontal inner cortex contours passing the furcation of the first molar paralleled to the lower occlusal plane. The Generalized Procrustes Analysis (GPA) was applied to depict average contours and calculate the ratio and difference width of both upper and lower dental arches in different positions. Variations of the basal bone morphology among individuals were revealed using Principal Component Analysis (PCA).

**Results:**

The width discrepancy occurred at 7-7 segment (male: upper 65.62 mm and lower 68.81 mm and female: upper 62.98 mm and lower 68.38 mm) and the retromolar region (male: upper 64.67 mm and lower 71.96 mm and female: upper 62.34 mm and lower 71.44 mm). The ratio (*p* = 0.006) and difference value (*p* = 0.009) of 7-7 segment and the ratio of retromolar region (*p* = 0.044) differed in genders. Setting 2 mm overjet, the upper basal bone arch was wider than the lower by approximate 2 mm on both sides, except the second molar and retromolar region. According to PCA, the variation of basal bone arches appeared mainly at terminal segments.

**Conclusions:**

For both male and female, the bimaxillary basal bone matched except terminal segments. Mismatch of female bimaxillary posterior basal bone was more pronounced than male. The basal bone arches of male were wider and longer than that of female.

## 1. Introduction

As the supporting bones of teeth arch, basal bone arch shapes the dental arch [1, 2] and limits the boundary of tooth movement [3]. The cortex of alveolar bone is a vital tooth movement boundary during orthodontic treatment [4]. If tooth movement contacts with the endocortical bone, root resorption tends to occur [5, 6]. If it is detached from the outer cortical bone, the risk of gingival recession will be raised, compromising periodontal support tissue [7].

The definition of the basal bone border was still various [8–10]. The furcation, as a region to resist the loads incurred during mastication [11], was important in orthodontic treatment. In our study, we define “basal bone” as “the inner cortex contours of the upper and lower on the horizontal plane, which was passing the furcation of the maxillary or mandibular first molar root paralleled to the lower occlusal plane.”

Several studies have illustrated the characteristics of basal bone forms among individuals of different malocclusions and races [7, 12–15]. However, most of them focused on the buccal boundary from the incisor to the second molar [8–10]. So, there were a few studies that involved in the integral contours of the basal bone arches, including the lingual contours and the retromolar region. Therefore, it is necessary to acquire a comprehensive understanding of the bimaxillary basal bone contours including the retromolar region, to investigate the match between the upper and lower basal bone arches.

Hitherto, the reversible effects of extraction were still being concerned in clinic. Previous studies have revealed the possibility of upper airway dimension reduction [16–18], impaired oral motor functions [19], a lower myoelectric activity of the anterior, right and left suprahyoid muscles [20], and significant neuroplastic changes related to the ability to adapt to the altered oral conditions [21] after teeth extraction. Therefore, orthodontic clinicians tend to explore more methods to gain space without extraction. Recently, with the development of oral implantology and invisible orthodontics, molar distalization and maxillary expansion were applied more to gain space with fewer irreversible effects [7, 22]. However, previous studies paid more attention to the sagittal available space, e.g., retromolar space [23, 24], disregarding transverse limits, and the possible transverse discrepancy of retromolar space in bimaxillary. To minimize the risk of potential damage to molar roots and alveolar bone, the overall morphology of the basal bone, especially for the transverse retromolar region, should be illustrated.

Including laser scanning system, cast-analyzing software [13, 14], and beta function [15, 25] to provide curve fitting, various varieties of mathematical methods were applied to fit for basal arch forms. For average enclosed contour analysis, the Generalized Procrustes Analysis (GPA) suited more for shape registration and quantitive illustration [26], which was wide as a morphometric analysis approach for medical morphometric data [27, 28]. The Principal Component Analysis (PCA), which was widely applied in the evaluation of shape variation [29–31], used the dimensionality reduction technique to replace the original multiple variables and obtained the contribution rate of each principal component by calculating the score of the comprehensive principal component functions to evaluate the multivariate.

Therefore, in this study, the basal bone arches were indicated by the GPA, and its variations among individuals were evaluated by PCA.

The purpose of this study was to evaluate the match of average basal bone arches of the upper and lower in different genders with GPA and to clarify its variations among individuals with PCA. The specific objectives of this study were as follows: (1) to compare the differences of basal bone morphology in gender, (2) to illustrate the match of the basal bone of the upper and lower in different genders by GPA, and (3) to explore the variations of the basal bone morphology among individuals in different genders by PCA.

## 2. Materials and Methods

### 2.1. Design and Samples

This study was reviewed and approved by the ethics committee of Stomatology Hospital of Shandong University (No. 20210404), which included 100 young adults of the Department of Orthodontics (50 males and 50 females, range: 18–21 years; mean age: male 21.6 ± 1.0 years; female: 21.8 ± 1.1 years) from 2017 to 2019. An informed consent form was signed by each patient. Cone-beam computed tomography (CBCT) images were used to analyze the basal bone contours [32].

The inclusion criteria were as follows: (1) complete permanent dentition from the incisor to the second molar (including or excluding third molar); (2) mild spacing or crowding of each arch (≤3 mm); (3) alveolar bone level being above the furcation of the mandibular molar; and (4) no previous orthodontic treatment or maxillofacial surgery, no ectopic eruption teeth and impacted teeth of full dentition, no alveolar bone defect or lesion, no acute periodontitis, and no obvious asymmetry mandible.

The exclusion criteria were as follows: (1) defective dentition or ectopic eruption teeth or impacted teeth of full dentition, (2) crowding ≥ 3 mm or spacing ≥ 3 mm and incomplete supporting arch, (3) previous orthodontic treatment or maxillofacial surgery, (4) dentition with prosthetic crown or clinical crown form, (5) alveolar bone defect or lesion or acute periodontitis, and (6) obvious asymmetry mandible.

### 2.2. CBCT Protocols

The CBCT scans of all patients were acquired using a scanner (NewTom 5G, Quantitative Radiology, Italy) under the following conditionings: 110 kV, 7.33 mA, and4.8 seconds typical X-ray emission time; 18 × 16 cm field of view; and standard voxel size of 0.3 mm. The obtained digital images as DICOM files were imported into Dolphin Imaging (v.11.8, Chatsworth, USA).

**Figure 1:**
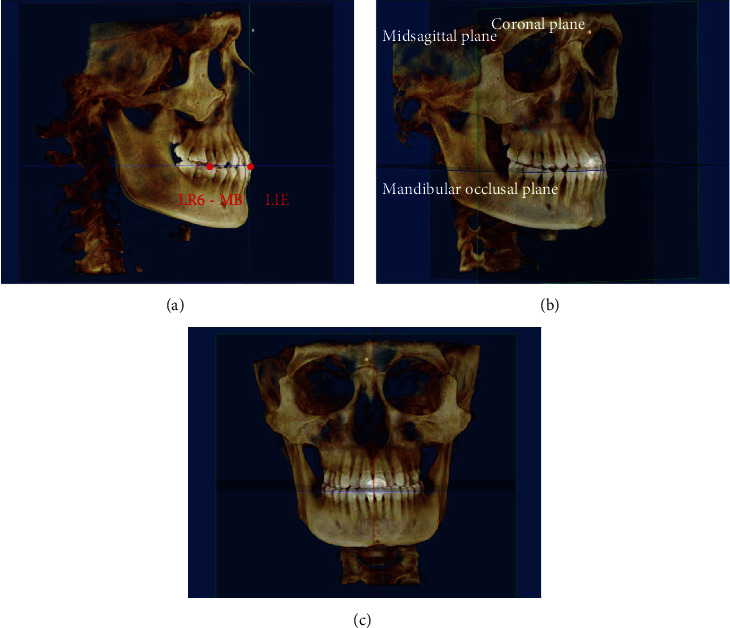
Reference planes and landmarks for reorientation of the 3D volumetric images. LIE: midpoint of the mandibular incisors' tips; LR6-MB: mesiobuccal cusp of the mandibular first molar.

Before the depicted contours or taking measurements, the 3D volumetric images were reoriented as follows (Figure 1): (1) The horizontal plane was adjusted to be parallel to the mandibular occlusal plane, connecting both mesiobuccal cusps of the mandibular first molar (LR6-MB) and the midpoint of the mandibular incisors' tips (LIE). (2) The sagittal plane was adjusted to be parallel to the median palatine plane, connecting anterior nasal spine (ANS) and posterior nasal spine (PNS), passing through LIE. (3) The coronal plane was set to be perpendicular to the sagittal plane and the horizontal plane.

The measured horizontal contours of the bimaxillary basal bone were obtained on the plane passing through the furcation of the first molar root of the maxilla and the mandible.

**Figure 2:**
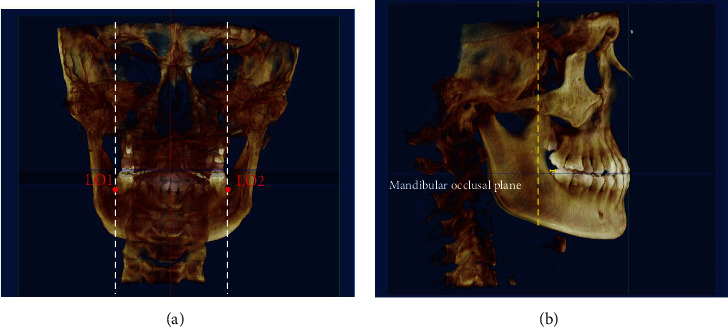
(a) Bilateral boundary of retromolar space in the coronal plane; LO1 and LO2: the outermost points of the last molar roots from lingual side. (b) Yellow line, posterior available space.

The cortical bone limitation of the retromolar region was decided by the lingual cortex of mandibular body [33]; thus, the retromolar region cortical limitation was identified in two planes. In the coronal plane, the bilateral boundary was the lines perpendicular to the horizontal plane, passing points LO1 and LO2, which were the outermost points of the last molar roots from lingual side (Figure 2(a)). In the sagittal plane, the available retromolar region was the distance from the vertical tangent of the ramus to the last teeth paralleled to the mandibular occlusal plane (Figure 2(b)).

**Figure 3:**
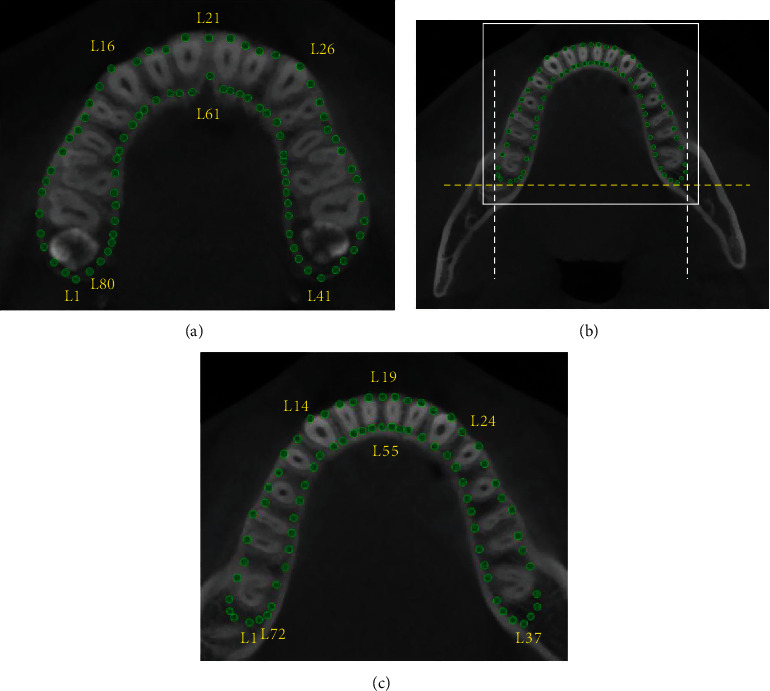
(a) 80 landmarks marked on inner cortical border image of the maxillary on the horizontal plane. (b) 72 landmarks marked on inner cortical border image of the mandible on the horizontal plane. White dotted line, bilateral boundary of retromolar space in the coronal plane and yellow dotted line, limits of retromolar space in the sagittal plane. (c) Close-up view of the white box in (b).

At the horizontal plane of the maxilla, Dolphin Imaging was used to mark along the inner cortical bone contour [5, 6], which was determined by 80 landmarks (Figure 3(a)), meanwhile 72 landmarks on that of the mandible (Figures 3(b) and 3(c)). All landmarks were averagely corresponding to the tooth positions of maxillary and mandibular basal bone (Tables 1 and 2).

### 2.3. Statistical Analysis Methods

The GPA was performed to acquire the bimaxillary average basal bone arches in different genders. Widths of basal bone in different tooth positions were calculated. Independent-sample *t*-test was performed to confirm whether the match of bimaxillary width differed in gender by SPSS (v.26.0, IBM, USA) if data were normally distributed. PCA was used to ascertain variations of basal bone morphological among individuals. After PCA, three principal factors were extracted to account for more than 50% of the cumulative proportion of the basal bone morphology variance among individuals in different genders. One examiner performed all measurements. To estimate reliability, twenty randomly selected subjects were reevaluated after one week. The intraclass correlation coefficient (ICC) showed high reliability (0.92 < ICC < 0.99). Data normality of variances was assessed by Shapiro-Wilk.

## 3. Results

### 3.1. Difference of Average Basal Bone Contours of Bimaxillary in Different Genders Using GPA

**Figure 4:**
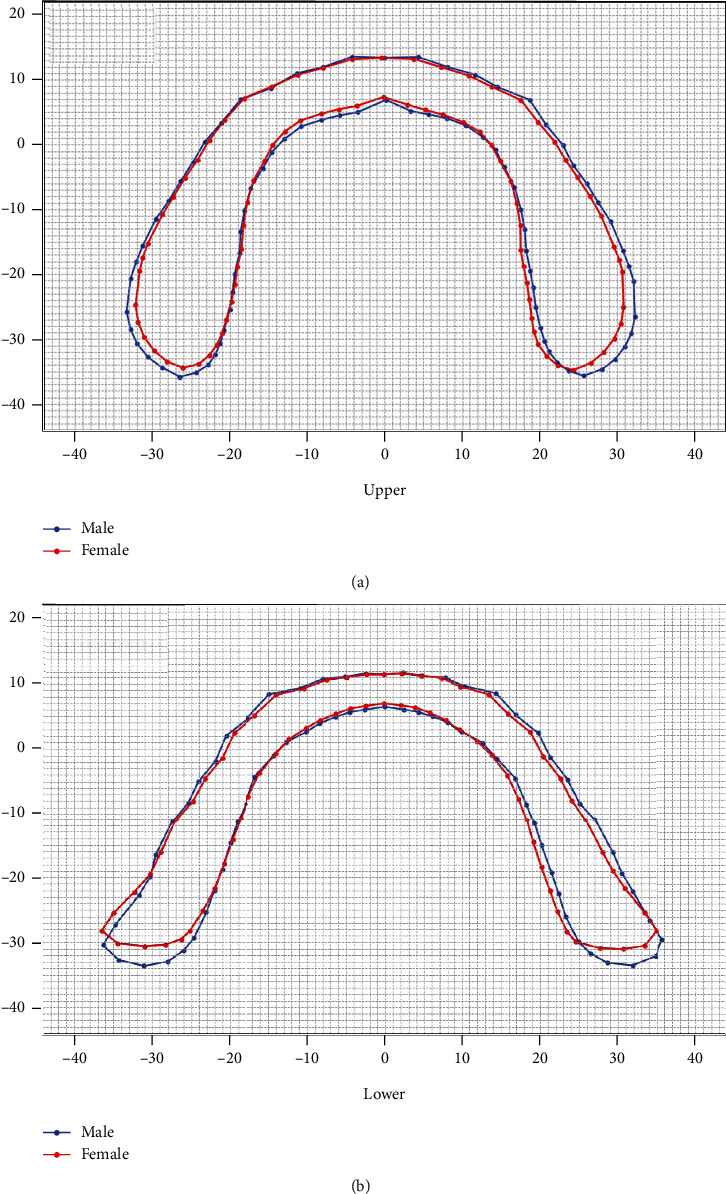
Basal bone contours of single jaw in different genders. One grid represented 1 mm. (a) Average upper basal bone contours in different genders. (b) Average lower basal bone contours in different genders.

The GPAs for average basal bone contours of bimaxillary in different genders are shown in Figure 4. Either in the maxilla or mandibular, the basal bone contours of the male were wider and longer than those of the female (Figure 4). The widths of the upper and lower basal bone of the male were, respectively, 2.64 mm and 0.52 mm larger than that of female. For length of the upper and lower, the basal bones of the male were, respectively, 1.07 mm and 2.64 mm larger than that of female (Table 3).

### 3.2. Match of Bimaxillary Basal Bone Arches in Different Genders Using GPA and Independent *t*-Test

Whether male or female, the matching tendencies of bimaxillary basal bone arches were similar. We calculated the width of basal bone in different tooth positions by GPA results. For the first molar, the upper landmarks for calculation were L9 and L33, and the lower landmarks were L7 and L31. For the second molar, the upper landmarks were L6 and L36, and the lower landmarks were L4 and L34. For the retromolar region, the upper landmarks were L5 and L37, and the lower landmarks were L3 and L35.  Table 4 shows that from the central incisor to the first molar, the widths of the upper basal bone were larger that of the lower one. The bimaxillary basal bone matched well, since the upper one was wider than the lower one, ranging from 3.12 mm to 8.09 mm for male and ranging from 3.28 mm to 8.30 mm for female. However, mismatch occurred at the second molar and the retromolar region, since there was width discrepancy. We used the ratio (upper/lower) and difference width (upper-lower) to describe the mismatch. For male, the widths of the upper second molar and retromolar region were, respectively, 3.19 mm and 7.30 mm shorter than the lower one. For female, the discrepancies were, respectively, indicated as 5.39 mm and 9.09 mm.

To explore whether the mismatch of posterior basal bone was different in gender, we applied independent-sample *t*-test after the data of 100 samples were tested as normal contribution. We found that ratio (U7-7/L7-7) (*p* = 0.006), difference width (U7-7-L7-7) (*p* = 0.009), and ratio (upper/lower retromolar region) (*p* = 0.044) were different in genders. The width discrepancy of the bimaxillary posterior basal bone for females (ranging from 5.38 mm to 9.07 mm) was more pronounced than males (ranging from 3.19 mm to 7.29 mm) (Table 5).

**Figure 5:**
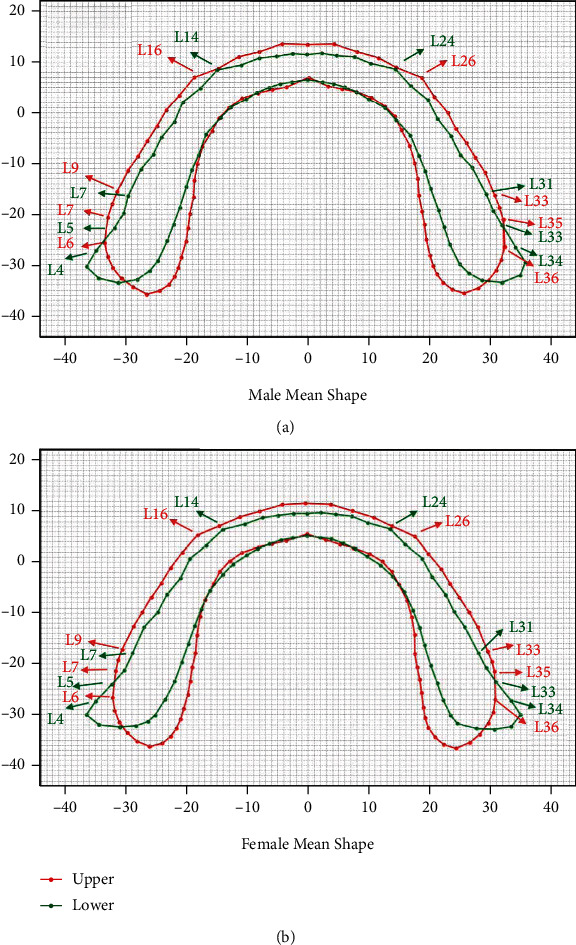
Average basal bone contours of bimaxillary in different genders. The upper one was put 2 mm above the lower one after adjustment. One grid represented 1 mm. (a) Male average basal bone contours after adjustment. (b) Female average basal bone contours after adjustment.

According to the normal overjet, the maxillary basal bone was adjusted to be 2 mm above the mandibular one at the midpoint of the upper incisor (Figure 5). We found that at the incisor segments (upper: red, L16-L26 and lower: green, L14-L24), the bicuspid segment, and the first molar segment (upper: red, L9-L33 and lower: green, L7-L31), the widths of the upper were approximately 2 mm larger than that of the lower on both sides. However, at the posterior segment, from the second molar to the retromolar space (upper: red, L6-L7 and L35-L36 and lower: green, L4-L5 and L33-L34), mismatch occurred, as for the closing-in two sides of the upper basal bone arch and the divergent two sides of the lower one (Figure 5).

### 3.3. PCA for Basal Bone Morphology Variations among Individuals of Male and Female

**Figure 6:**
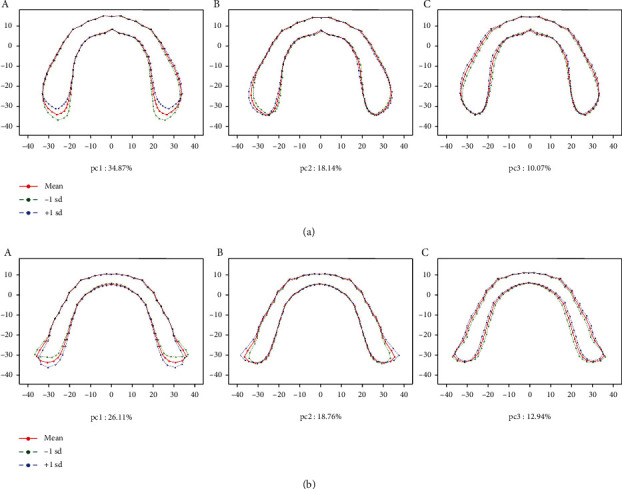
PCA for men's basal bone variance. (a) PCA result of men's maxillary basal bone variance. A, PC1: 34.87%; B, PC2: 18.14%; and C, PC3: 10.07%; (b) PCA result of men's mandibular basal bone variance. A, PC1: 26.11%; B, PC2: 18.76%; and C, PC3: 12.94%.

**Figure 7:**
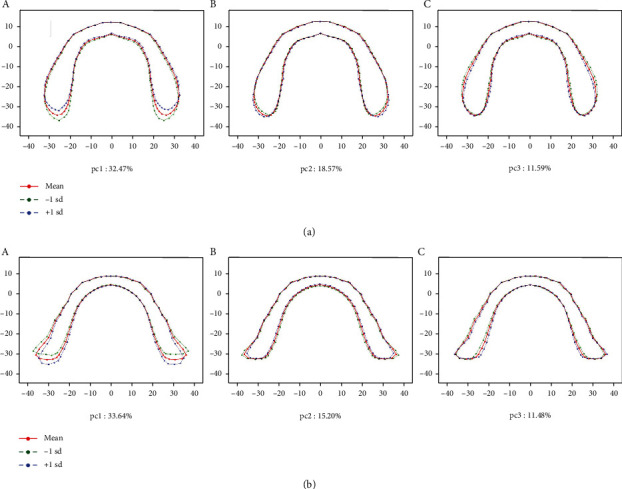
PCA for women's basal bone variance. (a) PCA result of women's maxillary basal bone variance. A, PC1: 32.47%; B, PC2: 18.57%; and C, PC3: 11.59%; (b) PCA result of women's mandibular basal bone variance. A, PC1: 33.64%; B, PC2: 15.20%; and C, PC3: 11.48%.

**Figure 8:**
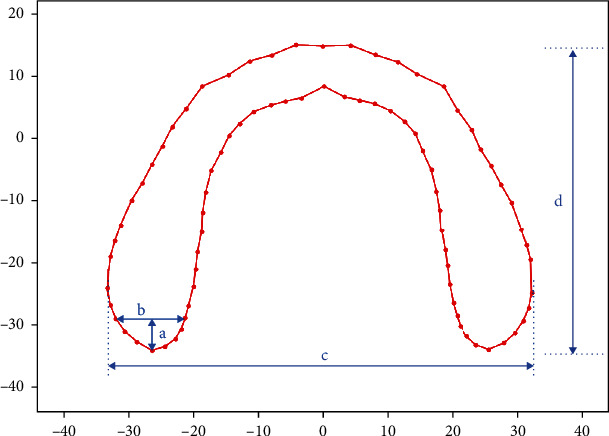
Characteristics of retromolar region. (a) Length of retromolar region. (b) Width of retromolar region. (c) Width of basal bone. (d) Length of basal bone.

The PCA results of basal bone morphology variances of males and females are shown in Figures 6 and 7 and Table 6. PCA accounted for more than 50% of the cumulative proportion of shape variance (Table 6). From PCA, we found that primary variations appeared at the posterior segment, especially at the retromolar region. The characteristics of retromolar regions were expressed using length and width. Vertical distance through the farthest point of the right side to the line represented the width stand for the length of the retromolar region (Figure 8(a)). Width of the retromolar region was the maximum distance of junctions with the retromolar region and the horizontal axis (Figure 8(b)). Besides, the width of basal bone represented the maximum distance of the bilateral sides along the horizontal axis (Figure 8(c)), while the length of basal bone was represented by the maximum distance of the bilateral sides along the vertical axis (Figure 8(d)).

For PCA of male upper basal bone morphology, principal component 1 (PC1) had an increasing length of the retromolar region at -1sd and had a decreasing length of the retromolar region at +1sd compared with the average bone morphology. Principal component 2 (PC2) had a decreased width of basal bone and had an opposite variation at +1sd compared to the average morphology. Principal component 3 (PC3) had a little narrower bicuspid segment of basal bone at -1sd while a little wider one at +1sd (Figure 6(a)).

For male lower basal bone morphology, PC1 had a decreased length of the retromolar region and an increased width of the basal bone at -1sd. PC2 had a decreased width of the retromolar region at -1sd. PC3 had a decreased width of the bicuspid basal bone segment at -1sd (Figure 6(b)).

For female upper basal bone morphology, PC1 and PC2 were similar to that of males. PC3 indicated little wider bicuspid basal bone segment at -1sd. (Figure 7(a)).

For female lower basal bone morphology, PC1 was similar to that of males. PC2 showed a wider retromolar region at -1sd. PC3 had a slightly wider bicuspid basal bone segment at -1sd (Figure 7(b)).

## 4. Discussion

The objective of this study was to illustrate the average basal bone arch morphology of the bimaxillary in different genders using the GPA and reveal match of the upper one and lower one. The basal bone morphology variations among individuals were illustrated by the PCA.

### 4.1. Basal Bone Arches Involved the Lingual and Retromolar Regions

No matter what methods were used in previous studies related to the basal bone, only the buccal contours were involved. Howes demonstrated the narrowest region of the alveolar bone, the basal bone, which was 8 mm below the marginal gingiva [8]. In 2000, LF Andrews and WA Andrews defined WALA ridge as a marginal border of the basal bone [3]. However, with the development of implantology, molar distalization, maxillary expansion, and other new nonextraction orthodontic strategies, the morphology of lingual alveolar bone should be paid more attention in case of fenestration and dehiscence, especially for molar distalization [34]. Therefore, our study involved the lingual boundary of the basal bones and defined cortical contours parallel to the mandibular occlusal plane and passing through the root furcation of the maxillary first molar and mandibular first molar as the contour of the basal bone. Tooth furcation, as the resistance center at the middle 1/3 of the teeth roots, served as the centroid of tooth roots. Third molar missing or impacted were not excluded since we set limitation of the posterior region. Cortical contours on the tooth furcation plane represented the safe moving range of tooth roots, and it involved the buccal and lingual contour enclosed lines including the retromolar region with the meaning of volume. This method was precise, convenient, and reliable.

### 4.2. The GPA for Irregular Structures

The GPA was an advanced geometric morphometric analysis to depict the average morphology of an irregular structure, such as the cross-sectional morphology of the mandible [26], the fibula [35], the unilateral coronal synostosis [36], and the corpus callosum [37], while making a quantified evaluation. The datasets were submitted for rotation and scaling to a common centroid, which help avoiding deviation of images and improving reliability. Therefore, this method was applied widely for clinical imaging analysis [35–37]. In our study, GPA was performed to illustrate the mean enclosed contours of bimaxillary basal bone including the retromolar region. Additionally, we added square grids to stand for the 1 mm ruler scale as background (Figure 4). It can provide an intuitive impression of basal bone differences in genders and in bimaxillary and quantify the morphology of the bimaxillary basal bones.

### 4.3. Transverse Mismatch of Bimaxillary Basal Bone Occurred at the Posterior Segments

Interesting finding was that the width of the upper arch was larger than that of the lower arch from the central incisor to the first molar segments while there was a discrepancy at the terminal segments (the second molar and the retromolar region) for the bimaxillary basal bone. As shown in Table 4, width of the upper basal bone was shorter than that of the lower at the second molar and the retromolar region. For male, at the second molar segment, the difference width was 3.19 mm, while the upper width was 65.62 mm, and the lower width was 68.81 mm. At the retromolar region, the difference width was 7.30 mm, while the upper width was 64.67 mm, and the lower width was 71.96 mm. For females, the difference widths were, respectively, 5.39 mm and 9.09 mm, which were larger than those of males.

### 4.4. Mismatch Tendencies Differed in Gender

The mismatch tendency of the posterior basal bone was different in gender. There were significant differences in width ratio (U7-7/L7-7) (*p* = 0.006), difference width (U7-7-L7-7) (*p* = 0.009), and width ratio (upper/lower retromolar region) (*p* = 0.044) (Table 5). Mismatch for female was more pronounced than that of male, which reminded us to pay more attention to female's posterior width coordination.

### 4.5. Match of Bimaxillary Basal Bones after Setting 2 mm Overjet

To get closer to a real normal bite, we supposed the overjet as 2 mm to adjust the upper basal bone 2 mm above the lower one (Figure 5). Then, we found that for both male and female, the segments from the incisor to the first molar matched well. However, the posterior region (from the second molar to the retromolar region) mismatched, the same as the ratio and difference of width of GPA results (Table 4). It reflected the transverse discrepancies of terminal segment, which may be caused by the morphology difference between bimaxillary basal bones. With the tooth movement in the terminal direction, two sides of the upper basal bone arch became closer, while the two sides of the lower became more divergent. This finding reminded us clinically, as a supplement for element I of Andrew's six elements [3].

### 4.6. PCA for Variations of Basal Bone Morphology among Individuals

PCA, as a useful method for analysis of multivariate variation such as irregular morphology, has been widely applied in analysis of dental crown [29], plant organs [30], facial parts [31], etc. to analyze the shape variation. Traditional research methods for basal bone forms used length and width to demonstrate variations among individuals [13, 14, 38], ignoring variations of multidirections. In this study, PCA was used to obtain the contribution rate of each principal component by calculating the score of the comprehensive principal component function (Figures 6 and 7). It can magnify the variation and reveal the regions of basal bones that differ in individuals with greater possibility.

PCA results indicated variations of basal bone arches among individuals. It explained more than 50% variations among subjects and indicated a variation of morphology at ±1sd. Furthermore, it illustrated that either for males or females, variations of basal bones occurred more at the terminal segments, while anterior segments were relatively stable among individuals. Combining the GPA results, it reminded us to pay more attention to the posterior segments.

### 4.7. Strengths and Limitations

This study was the first of its kind that investigated basal bones involving the lingual and retromolar regions. More clinicians realized the significance of anteroposterior available retromolar space to the achievement of molar distalization [24, 33, 39] with the development of nontooth extraction strategies [7, 22]. However, the results remind us that the limit of teeth compensate due to width mismatch of posterior segments should be given more attention, even though the sagittal retromolar space was enough, while for distalization, the upper basal bone was convergent and the lower basal bone was divergent. Therefore, buccal movement [40, 41] compensation of upper posterior teeth during molar distalization was restricted. Drooping palatal tips and deep Wilson curve should be avoided to prevent lateral movement interference.

Previous studies focused on the first molar when evaluating the width of the basal bone [42–44]. The results of this study reminded us that more evaluations of the transverse coordination of the second molar and the retromolar region between the upper and lower basal bone were necessary. Since the transverse width of the maxillary was often observed to be smaller than that of mandible in class III malocclusion [45, 46], we could deduce that while applying molar distalization of the mandibular dentition [47], the mismatch of the second molar and retromolar region basal bone may be more obvious. Therefore, in some circumstances, maxillary expansion or mandibular constrictor was necessary to some extent. To achieve a good match of the upper and lower basal bone, clinicians should be more cautious about the transverse match tendency of bimaxillary basal bone when designing schemes for molar distalization or evaluating width, especially for the terminal segments. This may also be applicable to forecast basal bone shape according to gender, avoiding root absorption during tooth movement. The study of basal bone variations among individuals will be needed in the future.

## 5. Conclusions

1. Generally, individual basal bones were different. On account of the closing-in two sides of the upper basal bone arch and the divergent one of the lower, the bimaxillary basal bones matched except the terminal segments
2. The basal bone of the male was wider and longer than that of females
3. Mismatch of posterior basal bone differed in gender, and the mismatch of female bimaxillary posterior basal bone was more pronounced than males
4. Variations of basal bone among individuals occurred more at the retromolar region

## Figures and Tables

**Table 1:** Landmarks and definitions of points in maxillary basal bone.

L (landmark)	Definition
L1	Farthest point of right maxillary basal bone
L2-L5	Buccal retromolar space of right maxillary basal bone
L6-L8	The buccal point of second molar and buccal midpoint of second molar and first molar in right maxillary basal bone
L9-L11	The buccal point of first molar and buccal midpoint of first molar and second premolar in right maxillary basal bone
L12-L13	The buccal point of second premolar and buccal midpoint of second premolar and first premolar in right maxillary basal bone
L14-L15	The buccal contact point of first premolar and buccal midpoint of first premolar and canine in right maxillary basal bone
L16-L20	The buccal point from canine to central incisor in right maxillary “basal bone”
L21	The buccal forefront point of maxillary basal bone
L22-L26	The buccal point from central incisor to canine in left maxillary basal bone
L27-L30	The buccal midpoint of canine and first premolar and buccal point of second premolar in left maxillary basal bone
L31-L33	The buccal midpoint of second premolar and first molar and buccal point of first molar in left maxillary basal bone
L34-L36	The buccal midpoint of first molar and second molar and buccal point of second molar in left maxillary basal bone
L37-L40	Buccal retromolar space of left maxillary basal bone
L41	Farthest point of left maxillary basal bone
L42-L45	Lingual retromolar space of left maxillary basal bone
L46-L48	The lingual point of second molar and lingual midpoint of second molar and first molar in left maxillary basal bone
L49-L51	The lingual point of first molar and lingual midpoint of first molar and second premolar in left maxillary basal bone
L52-L55	The lingual point of second premolar and lingual midpoint of first premolar and canine in left maxillary basal bone
L56-L60	The lingual point from canine to central incisor in left maxillary basal bone
L61	The lingual forefront point of maxillary basal bone
L62-L66	The lingual point from central incisor to canine in right maxillary basal bone
L67-L70	The lingual midpoint of canine and first premolar and lingual point of second premolar in right maxillary basal bone
L71-L73	The lingual midpoint of second premolar and first molar and lingual point of first molar in right maxillary basal bone
L74-L76	The lingual midpoint of first molar and second molar and lingual point of second molar in right maxillary basal bone
L77-L80	Lingual retromolar space of right maxillary basal bone

**Table 2:** Landmarks and definitions of points in mandibular basal bone.

L (landmark)	Definition
L1	Farthest point of right mandible basal bone
L2-L3	Buccal retromolar space of right mandible basal bone
L4-L6	The buccal point of second molar and buccal midpoint of second molar and first molar in right mandible basal bone
L7-L9	The buccal point of first molar and buccal midpoint of first molar and second premolar in right mandible basal bone
L10-L11	The buccal point of second premolar and buccal midpoint of second premolar and first premolar in right mandible basal bone
L12-L13	The buccal contact point of first premolar and buccal midpoint of first premolar and canine in right mandible basal bone
L14-L18	The buccal point from canine to central incisor in right mandible basal bone
L19	The buccal forefront point of mandible basal bone
L20-L24	The buccal point from central incisor to canine in left mandible basal bone
L25-L28	The buccal midpoint of canine and first premolar and buccal point of second premolar in left mandibular basal bone
L29-L31	The buccal midpoint of second premolar and first molar and buccal point of first molar in left mandibular basal bone
L32-L34	The buccal midpoint of first molar and second molar and buccal point of second molar in left mandibular basal bone
L35-L36	Buccal retromolar space of left mandibular basal bone
L37	Farthest point of left mandibular basal bone
L38-L39	Lingual retromolar space of left mandibular basal bone
L40-L42	The lingual point of second molar and lingual midpoint of second molar and first molar in left mandibular basal bone
L43-L45	The lingual point of first molar and lingual midpoint of first molar and second premolar in left mandibular basal bone
L46-L49	The lingual point of second premolar and lingual midpoint of first premolar and canine in left mandibular basal bone
L50-L54	The lingual point from canine to central incisor in left mandibular basal bone
L55	The lingual forefront point of mandibular basal bone
L56-L60	The lingual point from central incisor to canine in right mandibular basal bone
L61-L64	The lingual midpoint of canine and first premolar and lingual point of second premolar in right mandibular basal bone
L65-L67	The lingual midpoint of second premolar and first molar and lingual point of first molar in right mandibular basal bone
L68-L70	The lingual midpoint of first molar and second molar and lingual point of second molar in right mandibular basal bone
L71-L72	Lingual retromolar space of right mandibular basal bone

**Table 3:** Width and length of basal bone of GPA results in different genders.

	Maxillary basal bone (mm)		Mandibular basal bone (mm)	
	Width	Length	Width	Length
Male	65.62	49.30	71.96	45.29
Female	62.98	48.23	71.44	42.65
Difference width	2.64	1.07	0.52	2.64

Note. Values are mean of the Generalized Procrustes Analysis (GPA) result.

**Table 4:** Width of basal bone in different tooth positions of GPA result.

	Male				Female			
	Upper (mm)	Lower (mm)	Ratio (U/L)	Difference value (U-L) (mm)	Upper (mm)	Lower (mm)	Ratio (U/L)	Difference value (U-L) (mm)
1-1	8.50	4.74	1.79	3.76	8.00	4.52	1.77	3.48
2-2	23.01	15.64	1.47	7.37	22.00	14.70	1.50	7.30
3-3	37.41	29.31	1.28	8.09	35.78	27.48	1.30	8.30
4-4	46.27	40.30	1.15	5.97	44.64	38.28	1.17	6.37
5-5	52.45	47.60	1.10	4.84	50.70	45.92	1.10	4.78
6-6	61.98	58.85	1.05	3.12	60.21	56.93	1.06	3.28
7-7	65.62	68.81	0.95	-3.19	62.98	68.38	0.92	-5.39
Retromolar region	64.67	71.96	0.90	-7.30	62.34	71.44	0.87	-9.09

**Table 5:** Width of the second molar and retromolar region of 100 samples in different genders.

	Male (*N* = 50)	Female (*N* = 50)	*t* value	*p* value
Upper 7-7 width	65.48 ± 2.18	62.86 ± 1.86	-6.44	0.000^∗∗∗^
Lower 7-7 width	68.67 ± 3.37	68.24 ± 3.84	-0.59	0.555
Ratio (U7-7/L7-7)	0.96 ± 0.06	0.92 ± 0.06	2.79	0.006^∗∗^
Difference width (U7-7-L7-7)	−3.19 ± 3.19	−5.38 ± 4.09	2.66	0.009^∗∗^
Upper retromolar region width	64.53 ± 2.38	62.23 ± 1.97	-5.26	0.000^∗∗∗^
Lower retromolar region width	71.82 ± 4.39	71.29 ± 4.06	-0.62	0.536
Width ratio (upper/lower retromolar region)	0.90 ± 0.07	0.88 ± 0.06	2.04	0.044^∗^
Difference width (upper-lower retromolar region)	−7.29 ± 5.02	−9.07 ± 4.68	1.83	0.071

Dependent *t*-test. Note. Values are mean ± standard deviation. ^∗^Statistically significant for *p* < 0.05. ^∗∗^Statistically significant for *p* < 0.01. ^∗∗∗^Statistically significant for *p* < 0.001.

**Table 6:** Proportion of PC explaining for basal bone shape variation.

	Male		Female	
	Maxillary basal bone	Mandibular basal bone	Maxillary basal bone	Mandibular basal bone
PC1	0.3487	0.2611	0.3247	0.3364
PC2	0.1814	0.1876	0.1857	0.1520
PC3	0.1007	0.1294	0.1159	0.1148
Sum	0.6308	0.5781	0.6263	0.6032

## Data Availability

The data that support the findings of this study are available from the corresponding author upon reasonable request.
